# Designing an optimal HIV programme for South Africa: Does the optimal package change when diminishing returns are considered?

**DOI:** 10.1186/s12889-017-4023-3

**Published:** 2017-01-31

**Authors:** Calvin Chiu, Leigh F. Johnson, Lise Jamieson, Bruce A. Larson, Gesine Meyer-Rath

**Affiliations:** 1 Health Economics and Epidemiology Research Office (HE2RO), Department of Internal Medicine, School of Clinical Medicine, Faculty of Health Sciences, University of the Witwatersrand, Johannesburg, South Africa; 2 Centre for Infectious Disease Epidemiology and Research (CIDER), University of Cape Town, Cape Town, South Africa; 3 Center for Global Health and Development, Department of International Health, Boston University, Boston, USA

**Keywords:** HIV, Modelling, Optimisation, Cost-effectiveness analysis, South Africa, Health economics

## Abstract

**Background:**

South Africa has a large domestically funded HIV programme with highly saturated coverage levels for most prevention and treatment interventions. To further optimise its allocative efficiency, we designed a novel optimisation method and examined whether the optimal package of interventions changes when interaction and non-linear scale-up effects are incorporated into cost-effectiveness analysis.

**Methods:**

The conventional league table method in cost-effectiveness analysis relies on the assumption of independence between interventions. We added methodology that allowed the simultaneous consideration of a large number of HIV interventions and their potentially diminishing marginal returns to scale. We analysed the incremental cost effectiveness ratio (ICER) of 16 HIV interventions based on a well-calibrated epidemiological model that accounted for interaction and non-linear scale-up effects, a custom cost model, and an optimisation routine that iteratively added the most cost-effective intervention onto a rolling baseline before evaluating all remaining options. We compared our results with those based on a league table.

**Results:**

The rank order of interventions did not differ substantially between the two methods- in each, increasing condom availability and male medical circumcision were found to be most cost-effective, followed by anti-retroviral therapy at current guidelines. However, interventions were less cost-effective throughout when evaluated under the optimisation method, indicating substantial diminishing marginal returns, with ICERs being on average 437% higher under our optimisation routine.

**Conclusions:**

Conventional league tables may exaggerate the cost-effectiveness of interventions when programmes are implemented at scale. Accounting for interaction and non-linear scale-up effects provides more realistic estimates in highly saturated real-world settings.

**Electronic supplementary material:**

The online version of this article (doi:10.1186/s12889-017-4023-3) contains supplementary material, which is available to authorized users.

## Background

Currently, over 35 million people are living with HIV globally. In 2013, there were 2.1 million new infections and 1.5 million AIDS-related deaths worldwide [1]. While UNAIDS maintains the aspirational aim of eliminating HIV by 2030 [2], this requires sustained commitment from international and domestic stakeholders. In the context of a shrinking funding landscape, where “flat lining [of budgets] is the new budget increase” [3], governments and donors alike are placing strong emphasis on pursuing allocative efficiency in HIV programming—selecting the mix of HIV programmes and interventions that produces a defined level of output at the lowest possible cost [4].

Some analysts have risen to the challenge of informing policy priorities by comparing the cost-effectiveness of single interventions, often using mathematical models. A recent combined analysis of 12 mathematical models examined the cost and cost-effectiveness of expanded treatment coverage and/or eligibility criteria for antiretroviral therapy (ART) [5]. The Bärnighausen, Bloom and Humair model compared medical male circumcision (MMC) against the provision of ART at current guidelines vs universal testing and treatment and applied it to several countries in sub-Saharan Africa [6]. Focusing on South Africa, Long et al. evaluated the cost-effectiveness of a range of interventions (ART, MMC, pre-exposure prophylaxis (PrEP), microbicides) both singularly and in combination [7]. Anderson et al. evaluated the impact at a subnational level of a similar package of interventions on the Kenyan HIV epidemic, targeting specific geographic locations that had high concentrations of female sex workers and men who have sex with men [8].

Going one step further, several models moved beyond comparing individual interventions and comprehensively assessed the cost and impact of entire national HIV programmes. Three models in particular—the AIDS Epidemic Model [9], GOALS [10], and Optima [11]—have been extensively applied to HIV/AIDS epidemics in a number of countries, projecting the cost and effectiveness of all interventions included in a country’s HIV programme under different scenarios. However, in a recent ‘fit-for-purpose’ assessment, the HIV Modelling Consortium noted that all three models assumed that interventions act independently, and that “interactions between programmes or technical and production efficiencies [could not] be adequately explored” [12].

In 2014, we were tasked by the South African government with providing the analytical framework for the country’s HIV Investment Case, which aimed to calculate the most cost-effective mix of interventions against HIV and inform relevant domestic and donor budgets. During that process, which has been described in detail elsewhere [13], we encountered challenges that necessitated the development of a custom optimisation routine that diverged from existing cost-effectiveness analytical techniques. After more than a decade of scale-up, the South African HIV response comprises a wide range of interventions, implemented at high levels of coverage [14]. These interventions often have interaction effects that challenge the assumption of independence between interventions which underpins conventional methods for cost-effectiveness analysis [15]- for example, scaling up any prevention intervention will likely reduce the need for treatment in later years, while scaling up treatment will reduce population HIV viral load and, by thus reducing HIV incidence, will reduce the need for prevention interventions The high baseline coverage levels of interventions also mean that the South African HIV programme is already quite saturated and interventions are likely to suffer from diminishing marginal returns (in other words, producing less additional impact for additional increases in coverage), thus producing non-linear scale-up effects that amplify the problem of interdependence of interventions.

South Africa is in the unique position of having a pre-existing, well-calibrated HIV transmission model that already takes into account those non-linear effects as well as interaction effects between interventions, the Thembisa model [16], which we extended with a custom cost model and optimisation routine. In our optimisation routine, we assessed the cost-effectiveness of interventions by iteratively adding the most cost-effective intervention onto a rolling baseline to which all previously selected interventions had already been added. Instead of defining a pre-specified linear relationship between cost, coverage, and outcome, we reran the epidemiological model at each stage. This allowed us to preserve the non-linear effects that occurred as interventions were scaled up and capture them in the subsequent cost-effectiveness analyses.

This paper describes the optimisation routine we developed for the South African HIV Investment Case and compares its results with those generated using conventional cost-effectiveness analysis methods to examine the incremental benefit of accounting for interaction effects between interventions and non-linear effects across scale up.

## Methods

### Interventions modelled for the Investment Case

Selection of intervention and coverage optionsAfter aprocess of evidence review and intervention selection which has been described elsewhere [17], we included 16 different HIV interventions with a demonstrated epidemiological impact in the Investment Case, and examined the impact of scaling each intervention up or down to up to 6 coverage levels: Baseline (BL) -2, −1, +1, +2, +3, and Feasible Maximum (FM), resulting in 101 distinct intervention-coverage options (Table 1). The Feasible Maximum represented an upper bound on the coverage level that could be reached by 2018/19 and was set at 70% for novel interventions and 95% for most existing interventions (Table 1). Notable exceptions to this rule are MMC and HIV counselling and testing (HCT) of the general population. The former was constrained by the model’s assumptions on demand for MMC, while the latter was defined based on government data. Scaling down of novel interventions with a baseline coverage of 0 was not considered; i.e., these do not have BL-2 and BL −1 options. Details regarding the unit costs, data sources used and assumptions made in parameterising each intervention-coverage option are provided in Additional file 1.

**Table 1:** List of interventions and coverage levels included in the optimisation routine

Intervention	Description	Coverage level							
		−2	−1	BL	+1	+2	+3	FM (2018/19)	
Antiretroviral therapy (ART) under current guidelines	Increase ART coverage while maintaining current eligibility criteria (CD4 < 500 and PMTCT B+ (triple ART initiation for life in all pregnant women))	✓	✓	-	✓	✓	✓	✓	95%
Universal treatment	Changing guidelines to allow for universal treatment (regardless of CD4 count) in addition to increasing ART coverage	✓	✓	✓	✓	✓	✓	✓	95%
Adult medical male circumcision (MMC)	Only unmarried men are assumed to get circumcised as a result of programmes that promote MMC as an HIV prevention strategy	✓	✓	-	✓	✓	✓	✓	550,000 circumcisions (model maximum)
Early infant male circumcision (EIMC)^a^	Circumcision of male infants in their first year of life	✓	✓	-	✓	✓	✓	✓	70%
Condom availability	This refers to distributing sufficient condoms to ensure that a specified proportion of sex acts (~24%) will be protected	✓	✓	-	✓	✓	✓	✓	95%
PrEP for female sex workers	Providing PrEP to sex workers only	-	-	-	✓	✓	✓	✓	70%
PrEP for young women	Providing PrEP to young women aged 15–24 only	-	-	-	✓	✓	✓	✓	70%
Prevention of mother to child transmission (PMTCT)		✓	✓	-	✓	✓	✓	✓	95%
Infant testing at birth	ART uptake in pregnant women	-	-	-	✓	✓	✓	✓	70%
Infant testing at 6 weeks		✓	✓	-	✓	✓	✓	✓	95%
HIV counselling and testing (HCT) of general population		✓	✓	-	✓	✓	✓	✓	18,153,000 tests (personal communication, NDoH)
HCT of sex workers		-	-	-	✓	✓	✓	✓	95%
HCT of adolescents	Dedicated HIV testing drives targeted at sex workers	-	-	-	✓	✓	✓	✓	95%
Social and behaviour change (SBCC) mass media campaign 1^b^	Dedicated HIV testing drives targeted at adolescents	-	✓	-	✓	✓	✓	✓	95%
SBCC mass media campaign 2	Message of reducing multiple sexual partners and increasing testing in adolescents	✓	✓	-	✓	✓	✓	✓	95%
SBCC mass media campaign 3	Message of increasing condom usage and self-efficacy	✓	✓	-	✓	✓	✓	✓	95%

^a^Although a novel intervention, the model assumed a non-zero baseline for EIMC [31]. We therefore retained the B-1 and B-2 coverage level scenarios in our analysis

^b^Since a number of organisations responsible for SBCC campaigns were involved in a government tender submission process at the time of analysis, we anonymised the campaigns in order to not unduly influence the tender process

### Cost and epidemiological modelling

We projected the epidemiological and cost impact of each intervention-coverage option over 20 years. We estimated the epidemiological impact in terms of life years saved relative to the West level 26 life table without applying any discounting [18]. Based on the model’s population estimates and data on the unit cost of each intervention, we projected the total cost of the entire HIV programme. Costs are evaluated from the provider perspective, presented undiscounted in constant 2014 US dollars (1USD = 11.05 ZAR). The details regarding how each specific intervention was costed and modelled in Thembisa have been described elsewhere [17].

These results were then used as inputs for a cost-effectiveness analysis using two different methods: firstly, the conventional league table method, and secondly using our optimisation routine that iteratively added the most cost-effective intervention onto a rolling baseline before evaluating all remaining options. We compared the results generated between the two methods to examine the incremental impact of our optimisation routine.

### Conventional league table method

We first analysed the cost-effectiveness of the interventions included in the Investment Case using the conventional league table method that is well established in existing literature [19–22]. Assuming independence between interventions, conventional league tables rank interventions in order of cost-effectiveness, allowing policy makers to decide which of the interventions on the list to implement, depending either on a budget constraint or a predetermined willingness-to-pay threshold. We constructed these league tables as follows.

First, we grouped different coverage levels of each intervention into separate categories. Second, we sorted the intervention-coverage level option by incremental cost in ascending order within each category. Third, we excluded options that were strongly dominated by another option in the same category—an option was strongly dominated if it was both less effective and more costly than an available alternative. Fourth, we calculated the incremental cost-effectiveness-ratio (ICER) of each successive option using the immediate intervention above it in the list as the baseline, and removed interventions that were weakly dominated.^1^ Fifth, we selected the intervention with the highest ICER that was still below the designated willingness to pay threshold. For the current analysis, in order to keep the list of interventions comparable across the two methods, we did not apply a budget constraint, which meant that the option with the highest ICER, typically the highest coverage level option (the feasible maximum), was chosen. Lastly, we combined the selected intervention in each of the mutually exclusive categories into a single league table, and ranked them in ascending order of cost-effectiveness ratio (CER) over baseline.

The fact that these CERs are calculated by comparing the impact of the intervention on baseline implicitly assumes that an intervention’s effectiveness is independent of what other interventions have already been implemented.

### Optimisation routine

Given that the South African HIV programme already consists of a wide range of interventions, we hypothesised that interaction effects between interventions were important and would challenge the assumption of independence between interventions that underlines the conventional league table method. Given that many interventions are also already implemented at high levels of coverage [14], we hypothesised that further increasing coverage in this saturated environment would have diminishing marginal returns, ie, increasingly less impact for every increment in coverage. This means that ICERs generated from conventional cost-effectiveness analyses are likely to overstate the impact of interventions—the same life year cannot be saved twice through two different interventions—and lead to overly optimistic policy decisions.

We therefore added a custom costing and optimisation component to Thembisa in order to preserve the non-linear scale-up effects inherent in the transmission model and produce more realistic cost-effectiveness results. First, we projected the cost and effect of the HIV programme at baseline. We then calculated the incremental cost and effect (in terms of life years saved) over baseline of each intervention-coverage level option. Next, we conducted a pairwise comparison between all interventions and filtered out all strongly dominated interventions.

We then ranked the remaining options by their ICER over baseline. The option with the lowest ICER was the most cost-effective. However, we also ranked between competing cost-saving options, which conventional cost-effectiveness analysis does not allow for since cost-saving options have negative ICERs that are not interpretable. In order to rank these cost-saving interventions, we ranked them both by life years saved (in descending order), and incremental cost (in ascending order). We then summed these two ranks and chose the intervention that had the lowest combined rank, representing the most effective and cost-saving intervention in aggregate. After choosing the most cost-effective intervention, we removed all lower coverage level options of the chosen intervention from the remaining pool.

In order to take into account the possibility of diminishing marginal returns as a result of an increasingly saturated programme, we then modified the baseline to include the intervention-coverage level option that was found to be most cost-effective and re-evaluated the cost-effectiveness of the remaining options using the same algorithm relative to this new baseline. We repeated this process iteratively until all intervention-coverage level options were exhausted (either because they had been selected and added to the baseline, or because they had been excluded from the analysis based on the algorithm). The order in which intervention-coverage level options were added to the baseline represents the relative cost-effectiveness of each intervention, and allows us to construct a ranking comparable with those from conventional league table analyses.

**Fig. 1:**
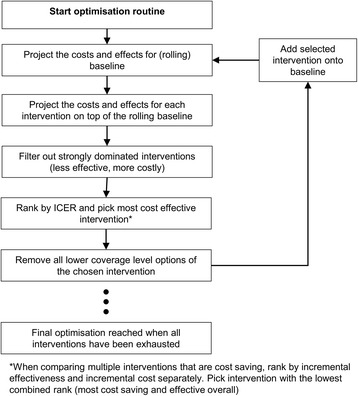
Optimisation algorithm and decision rules

Figure 1 illustrates this process.

## Results

**Fig. 2:**
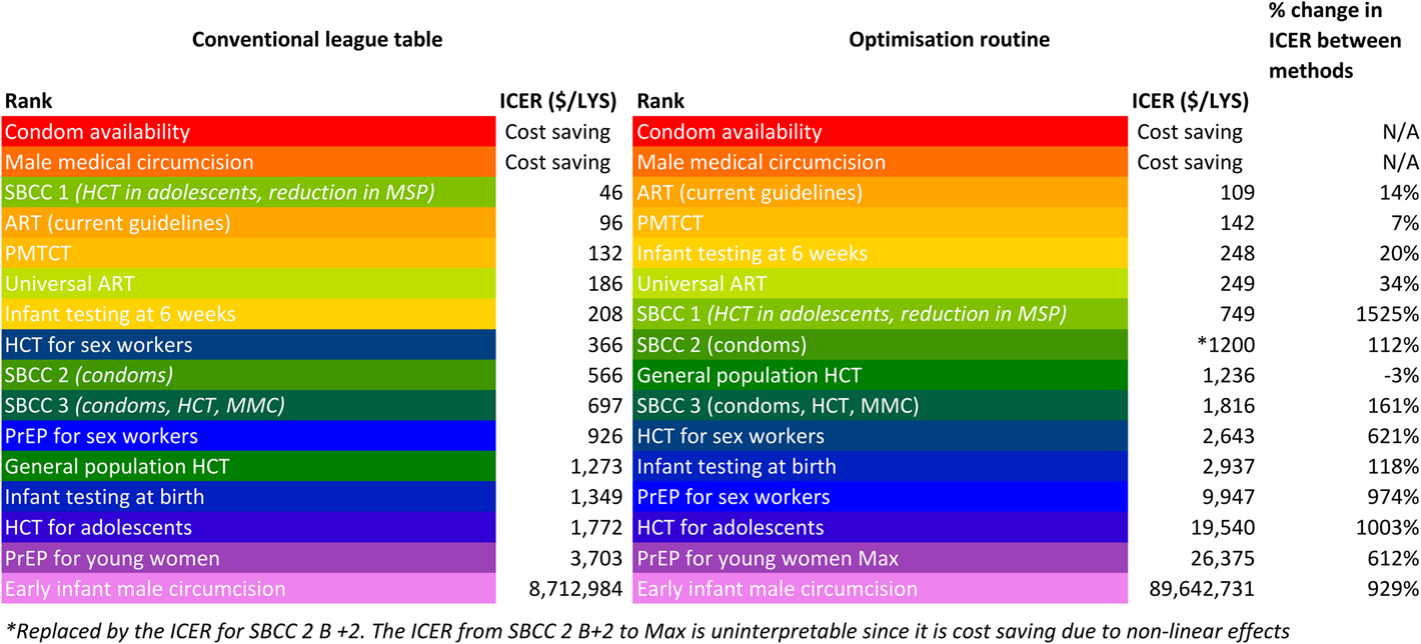
Comparison of ICERs and ranks between methods

Figure 2 summarises the ICERs and rank of each intervention’s feasible maximum coverage level option in a league table for each of the two cost-effectiveness analysis methods, in descending order of cost-effectiveness.

### Conventional league table method

Using the conventional league table method, scaling up condom availability and medical male circumcision were the most cost-effective options. The model in fact suggests that they were cost-saving overall, since both interventions prevent significant numbers of new infections which translate into savings in treatment costs and net overall savings. These were followed by social and behaviour change campaign (SBCC) 1 (a mass media campaign with a message of encouraging testing and discouraging multiple partners) and scaling up ART under current guidelines and prevention of mother to child transmission (PMTCT). Towards the bottom of the list, interventions with relatively poor cost-effectiveness over 20 years included HIV testing for adolescents, PrEP for young women, and early infant male circumcision.^2^ See Additional file 1 for the life years saved, total and incremental cost of each intervention using the league table method.

### Optimisation routine

The order of interventions in the league table using our optimisation routine was similar to the conventional league table method (Fig. 2), with some key exceptions. SBCC campaign 1, HCT for sex workers, and PrEP for sex workers were amongst the interventions that experienced the greatest percentage increase in the magnitude of their ICER, and were subsequently ranked significantly lower on the league table. Once the highly effective and cost-effective prevention interventions of condom distribution and male medical circumcision were implemented, and ART was scaled up to its feasible maximum coverage level (therefore producing a significant prevention benefit on its own [23]), the marginal impact of further prevention interventions was diminished.^3^ This suggests that conventional methods of economic evaluation are likely to overestimate the cost-effectiveness of interventions lower down a league table due to an inadequate consideration of diminishing marginal returns.

Focusing on the difference in magnitudes of the ICERs of each intervention between the two methods, we found that all interventions were less cost-effective (i.e. more costly and/or less effective) when evaluated against a stacked baseline of interventions with high coverage levels. Restricting our analysis to interventions with positive ICERs, the ICERs calculated using the optimisation routine were on average 437% higher than their counterparts under the conventional league table method. This shows substantial diminishing marginal returns to investment since interaction effects between interventions become more significant as the HIV response becomes increasingly saturated.

Table 2 presents the ranking and ICERs of all the intervention-coverage options included in the optimisation routine, allowing us to trace the order in which interventions were successively added to the baseline package of interventions. This is informative because the order in which different coverage levels of a given intervention are picked by the optimisation routine is suggestive of the strength of the recommendation.

**Table 2:** ICER league table using iterative optimisation routine

Intervention	Total cost over 20 years (2014 USD)	Life years saved over rolling baseline	Incremental cost over rolling baseline (2014 USD)	ICER (Cost/LYS)	Final rank
Baseline	52,533,337,028	-	-	-	-
Condom availability Max	51,022,627,998	3,899,254	−1,510,709,029	Cost-saving	1
MMC Max	51,014,858,122	951,825	−7,769,876	Cost-saving	2
SBCC1 B + 1	51,026,339,070	320,591	11,480,948	36	Superseded
ART (current guidelines) Max	51,243,805,676	2,004,044	217,466,606	109	3
PMTCT Max	51,314,427,752	497,889	70,622,076	142	4
Infant testing at 6 weeks B + 3	51,343,303,114	116,406	28,875,362	248	Superseded
Infant testing at 6 weeks Max	51,352,292,109	36,216	8,988,995	248	5
Universal ART Max	52,658,690,554	5,241,734	1,306,398,445	249	6
General population HCT B-2	52,002,826,154	−1,313,947	−655,864,400	499	Superseded
General population HCT B-1	52,338,752,835	738,355	335,926,681	455	Superseded
SBCC1 B + 2	52,406,222,629	111,661	67,469,794	604	Superseded
General population HCT B + 1	53,032,457,662	1,012,981	626,235,033	618	Superseded
SBCC1 B + 3	53,098,136,663	90,853	65,679,001	723	Superseded
SBCC1 Max	53,165,119,118	89,429	66,982,455	749	7
General population HCT B + 2	53,462,915,656	349,206	297,796,538	853	Superseded
General population HCT B + 3	53,755,702,602	285,671	292,786,946	1025	Superseded
SBCC2 B + 2	53,778,290,385	18,820	22,587,782	1200	Superseded
SBCC2 Max	53,777,167,130	37,516	−1,123,255	Cost-saving	8
General population HCT Max	54,066,875,384	234,391	289,708,254	1236	9
SBCC3 B-3	53,986,493,432	−50,161	−80,381,952	1602	Superseded
SBCC3 Max	54,236,500,801	137,678	250,007,369	1816	10
HCT for sex workers Max	54,277,669,534	15,576	41,168,734	2643	11
Infant testing at birth B + 2	54,547,672,527	91,981	270,002,992	2935	Superseded
Infant testing at birth B + 3	54,686,528,178	47,297	138,855,651	2936	Superseded
Infant testing at birth Max	54,817,666,909	44,648	131,138,732	2937	12
PrEP for sex workers Max	55,022,116,749	20,554	204,449,839	9947	13
Testing adolescents B + 1	55,538,796,938	31,920	516,680,189	16,187	Superseded
Testing adolescents B + 2	56,378,936,369	48,428	840,139,431	17,348	Superseded
Testing adolescents B + 3	57,309,365,304	48,915	930,428,935	19,021	Superseded
Testing adolescents Max	57,956,976,545	33,142	647,611,241	19,540	14
PrEP for young women Max	68,668,523,964	406,120	10,711,547,418	26,375	15
Early infant male circumcision B + 1	68,755,214,097	2	86,690,134	43,345,067	Superseded
Early infant male circumcision Max	69,024,142,290	3	268,928,193	89,642,731	16

While the model selected the maximum coverage level ahead of all other coverage levels in the cases of condom availability and medical male circumcision, the model recommended scaling up SBCC campaign 1 more incrementally. Scaling up SBCC campaign 1 to Baseline +1 was the most cost-effective (non-cost-saving) intervention, but the model only suggested further scale-up to maximum coverage once PMTCT, ART at current guidelines, and infant testing at 6 weeks had already been scaled up. A similar phenomenon was observed in the case of general population HCT, whose scaled-up coverage levels were interspersed between different coverage levels of SBCC campaigns 1 and 2. This suggests that there are non-linearities in the incremental effectiveness as interventions are scaled up, which implies the existence of an optimum coverage level in terms of cost-effectiveness, after which further scale-up would lead to diminishing marginal returns.

**Fig. 3:**
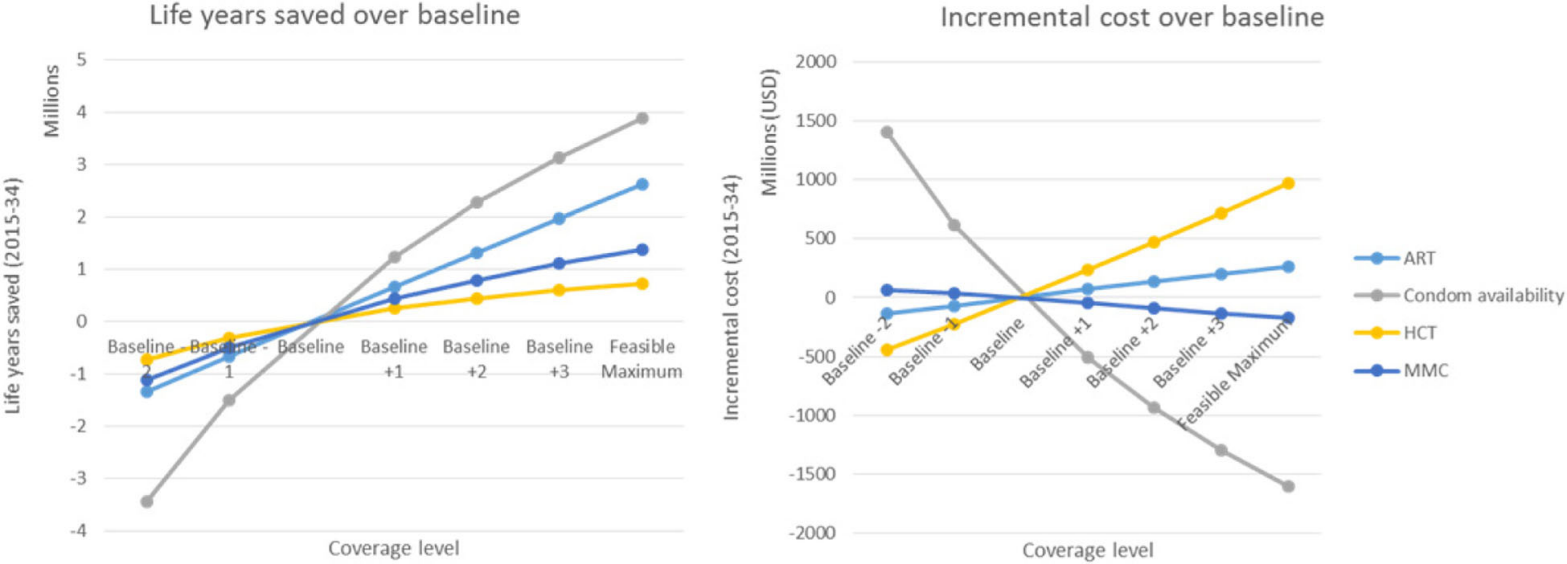
Life years saved and incremental cost over baseline for select intervention-coverage combinations

To illustrate this further, Fig. 3 plots the incremental cost and life years saved over baseline of equally spaced coverage levels of four selected interventions. For each of the four interventions, effectiveness exhibits diminishing marginal returns, providing evidence for non-linear scale-up effects.

## Discussion

Comparing our novel optimisation routine against conventional cost-effectiveness analysis methods using interventions included in the South African Investment Case as a case study, we found substantial gains in analytical precision from our methodology.

Firstly, our custom optimisation routine provided more realistic ICERs that accounted for interaction effects, resulting in diminishing marginal returns to scale. The magnitude of the differences in the ICERs between the two methods was non-trivial (437%), with the optimisation method especially penalising prevention interventions once other highly effective and cost-effective prevention interventions had already been taken to scale (condom distribution and male medical circumcision). This is particularly important in a relatively saturated environment like South Africa, especially since funding is likely to be limited in the future. Our consideration of interaction effects extends the broader literature on generalised cost-effectiveness analysis [15, 24], while the concrete examples of non-linearity in the relationship between coverage and outcomes found in our analysis contribute to the ongoing discussion over non-linear functions in the modelling the impact of diseases and interventions [25, 26].

Secondly, our approach of adding a custom optimisation routine onto a pre-existing, country-specific epidemiological model contrasted with the established approach of using integrated HIV epidemiological and costing models. We approached the same task of pursuing allocative efficiency by starting from a well-calibrated, context-specific epidemiological model and adding costing and cost-effectiveness evaluation to it subsequently. Our approach illustrated the benefit of drawing on the available detail of a well-parameterised epidemiological model, instead of a cost-effectiveness model designed to simplify the epidemiology for costing purposes. This meant we were able to avoid common practices such as linearising non-linear mechanisms.

Our optimisation routine is subject to several key limitations. Firstly, there is limited scope for uncertainty analysis in our optimisation model. The rank order of interventions could vary significantly as uncertainty bounds around the ICERs are likely to overlap. While adopting methods such as stochastic league tables [27, 28] may provide an upper and lower bound to the cost-effectiveness estimates, doing so requires computing all possible intervention combinations, which limits the benefits of a simplifying algorithm in the first place.

Secondly, our optimisation routine is limited to using cost per life years saved as the optimand (though it can also accommodate cost per HIV infections averted). This does not allow for optimisation based on specific epidemiological targets such as minimising incidence or mortality. Furthermore, our routine does not allow for optimisation based on multiple criteria, such as efficiency and equity [29]. Further research may be targeted towards conducting optimisation using a set of different criteria, individually weighted to reflect policy priorities, and collecting data to inform these relative weights. Thirdly, our optimisation routine is restricted by the coarseness of our pre-specified discrete coverage levels. Despite improving on the conventional league table method, our routine may still be detecting a local optimum, rather than the global maximum that we seek.

Fourthly, our algorithm cannot be considered as “optimisation” in the strictest sense of the word. Although we believe it to be a reasonable approximation, iteratively adding the most cost-effective option onto the baseline does not necessarily guarantee the globally optimal bundle of interventions, since it could theoretically be beneficial to remove certain options later on in the routine in favour of others. This is a classic case of the knapsack problem [30] which can only be overcome using a more complex optimisation routine that requires the model structure to be fully mathematically defined and differentiable, as in the case of Optima [11]. This, however, might come at the cost of sacrificing non-linearities and as such represents an analytical trade-off. Fifthly, the optimisation routine remains both computationally and time intensive. The conventional league table method may provide adequately accurate rankings between interventions in many analytical situations—when the scale and range of interventions in the HIV programme are limited, and the independence assumption is reasonably satisfied. Depending on the specific policy question at hand, the potential gains provided by the optimisation routine may not justify the additional computational requirements, especially in the absence of an existing detailed epidemiological model.

Lastly, our optimisation routine’s ability to generate more realistic ICER comes at a cost of the external validity of our results, since the cost-effectiveness estimates of each intervention in the league table is conditional on having implemented all of the interventions above. Since policy makers often pick and choose interventions for reasons other than maximising cost-effectiveness, any deviations from the rolling baseline will render the cost-effectiveness estimates inaccurate and might provide a false sense of precision.

## Conclusions

Conventional league table methods for cost-effectiveness analyses may exaggerate the cost-effectiveness of interventions when programmes are implemented at scale, such as in the context of the South African HIV programme. This is because the assumption of independence between interventions is challenged, and intervention scale-up often leads to diminishing marginal returns to an investment. We developed an optimisation routine that iteratively added the most-cost-effective intervention onto a rolling baseline to obtain more realistic ICERs and rank orders for interventions in highly saturated real-world settings. The results of our optimisation routine provided decision makers within the South African Department of Health and Treasury with reliable data on the relative cost effectiveness of a vast range of HIV interventions in such a setting, guiding decisions on targets and budgets for the next years and helping to increase the allocative efficiency of the country’s HIV expenditure.

## Additional file

Additional file 1: Technical appendix. (PDF 356 kb)

## Abbreviations

AIDS: Acquired immune deficiency syndrome
ART: Antiretroviral therapy
BL: Baseline
CER: Cost-effectiveness ratio
EIMC: Early infant male circumcision
FM: Feasible maximum
HCT: HIV counselling and testing
HIV: Human immunodeficiency virus
ICER: Incremental cost effectiveness ratio
LYS: Life years saved
MMC: Medical male circumcision
PMTCT: Prevention of mother to child transmission
PrEP: Pre-exposure prophylaxis
SBCC: Social and behaviour change campaign
UNAIDS: Joint United Nations Programme on HIV/AIDS
US: United States
USAID: United States Agency for International Development
USD: United States dollar
ZAR: South African Rand

## Footnotes

1 A is weakly dominated by B if in a rank order of A, B and C by incremental cost, B has an ICER that is higher than both A and C.

2 Similar to previous analyses we found that interventions targeted at infants were less cost-effective over a modelling period of 20 years, since the majority of prevention benefits in this population are realised only after the end of the modelling period.

3 While general population HCT may look like an exception, we believe that it only moved up the league table in relative terms (the other prevention interventions went down the league table), and because its unit cost was lower than HCT for other sub-populations, which made its cost-effectiveness more robust to changes in effectiveness.
