# Supplementary material for: Designing an optimal HIV programme for South Africa: Does the optimal package change when diminishing returns are considered?
Source: BMC Public Health. 2017 Jan 31;17:143. doi: 10.1186/s12889-017-4023-3 (PMC5282636; doi:10.1186/s12889-017-4023-3)
Supplement: Additional file 1: — Technical appendix. (PDF 356 kb) [file 12889_2017_4023_MOESM1_ESM.pdf]

Technical appendix

for the paper "*Designing an optimal HIV programme for South Africa: Does the optimal package change when diminishing returns are considered?*" by Calvin Chiu, Leigh F. Johnson, Lise Jamieson, Bruce A. Larson and Gesine Meyer-Rath

A: Data sources and model assumptions regarding the selection of intervention-coverage options

For all epidemiological projections, we used a pre-existing, well-calibrated HIV transmission model that accounts for non-linear effects of scale up. The Thembisa model is a deterministic compartmental model of the South African HIV epidemic maintained by the Centre for Infectious Disease Epidemiology and Research at the University of Cape Town [1].

Coverage levels were specified by linearly interpolating between the baseline and feasible maximum coverage levels. Coverage levels below baseline (minimum coverage) were determined by extrapolating the above linear trend to below baseline levels. Baseline coverage levels were informed by model outputs on the status quo based on the epidemiological model's fitting process [1], apart from the three social behaviour change communication campaigns whose coverage levels were informed by the most recent National HIV Communication Survey [2].

For existing interventions, we assumed a feasible maximum coverage of 95% by 2018/19, apart from medical male circumcision (MMC) and HIV counselling and testing (HCT). For MMC, the model reached a maximum of 550,000 circumcisions per annum in 2018/19 given inherent assumptions about demand and need for MMC. For HCT, we chose an upper limit of 18 million tests per annum after consultation with the National Department of Health, after taking current programmatic constraints into consideration. For all interventions not already introduced into the South African HIV programme, we assumed a feasible maximum coverage of 70% by 2018/19 given likely programmatic constraints to scaling up further in the short term.

B: Summary of unit costs by intervention

Intervention	Description	Unit costs (ZAR 2015)
Antiretroviral therapy (ART) under current guidelines	Increase ART coverage whilst maintaining current eligibility criteria (CD4<500 and PMTCT B+ (triple ART initiation for life in all pregnant women))	R 3,665 per patient year (adults, 2014/5)[1]
Universal treatment	Changing guidelines to allow for universal treatment (regardless of CD4 count) in addition to increasing ART coverage	R 3,412 per patient year (children, 2014/5)
Adult medical male circumcision (MMC)	Only unmarried men are assumed to get circumcised as a result of programmes that promote MMC as an HIV prevention strategy	R 1,431 per circumcision
Early infant male circumcision (EIMC)	Circumcision of male infants in their first year of life	R 605.10 per circumcision
Condom availability	This refers to distributing sufficient condoms to ensure that a specified proportion of sex acts will be protected	R 0.42 per condom distributed
PrEP for female sex workers	Providing PrEP to sex workers only	R 3,287 per patient year (first year)<br>R 2,548 per patient year (follow-up)

[1] Note that ART costs were calculated using the National ART Cost Model and vary over time.

Intervention	Description	Unit costs (ZAR 2015)
PrEP for young women	Providing PrEP to young women aged 15-24 only	R 3,105 per patient year (first year)<br>R 2,527 per patient year (follow-up)
Prevention of Mother To Child Transmission (PMTCT)	ART uptake in pregnant women	R 3,665 per patient year (adults, 2014/5)
Infant testing at birth		R 389.49 per test
Infant testing at 6 weeks		R 369.11 per test
HIV counselling and testing (HCT) of general population		R 87.76 per test (negative)<br>R 97.01 per test (positive)
HCT of sex workers	Dedicated HIV testing drives targeted at sex workers	
HCT of adolescents	Dedicated HIV testing drives targeted at adolescents	R 234.13 per test
Social and behaviour change (SBCC) mass media campaign 1	Message of reducing multiple sexual partners and increasing testing in adolescents	R 9.78 per person reached
SBCC mass media campaign 2	Message of increasing condom usage and self-efficacy	R 5.00 per person reached
SBCC mass media campaign 3	Message of increasing HIV testing, condom usage, condom self-efficacy, and MMC	R 2.74 per person reached

C: ICERs and rank order of interventions using the conventional league table method

Table 1: ICERs and rank order of interventions using the conventional league table method

Intervention	Total cost of intervention (2014 USD)	Incremental cost over baseline (2014 USD)	Life years saved over baseline	ICER (Cost/LYS)	Final rank
**Baseline**	52,533,337,028	-	-	-	-
Condom availability	51,022,627,998	-1,490,373,244	3,899,253	Cost saving	1
Male medical circumcision	52,369,215,443	-158,178,658	1,372,940	Cost saving	1
SBCC1	52,597,007,390	69,655,491	1,381,088	50	3
ART (current guidelines)	52,783,255,201	243,979,392	2,614,314	93	4
PMTCT	52,621,464,976	85,861,680	666,297	129	5
Universal treatment	54,386,572,548	1,812,436,748	9,940,779	182	6
Infant testing at 6 weeks	52,579,122,663	45,809,932	220,444	208	7
HCT for sex workers	52,573,269,976	39,772,123	109,004	365	8
SBCC2	52,582,120,264	49,297,508	86,165	572	9
SBCC3	52,643,609,707	110,906,185	158,211	701	10
PrEP for sex workers	52,683,046,199	149,079,539	161,601	923	11
General population HCT	53,441,216,557	909,747,251	713,141	1,276	12
Infant testing at birth	53,084,042,007	550,757,567	408,193	1,349	13
HCT for adolescents	55,842,339,089	3,308,036,903	1,867,487	1,771	14
PrEP for young women	63,288,723,746	10,699,314,344	2,904,336	3,684	15
EIMC	52,881,856,374	348,517,497	40	8,712,937	16

1. Johnson LF. THEMBISA version 1.0: A model for evaluating the impact of HIV/AIDS in South Africa. Cape Town: Centre for Infectious Disease Epidemiology and Research, University of Cape Town; 2014.

2. Johnson S, Kincaid D, Figueroa ME, Delate R, Mahlasela L, Magni S. The Third National HIV Communication Survey, 2012. . Pretoria: JHHESA, Lovelife, Soul City, 2012.
